# Text messaging as a community-based survey tool: a pilot study

**DOI:** 10.1186/1471-2458-14-936

**Published:** 2014-09-08

**Authors:** Tammy Chang, Weyinshet Gossa, Adam Sharp, Zachary Rowe, Lauren Kohatsu, Enesha M Cobb, Michele Heisler

**Affiliations:** Department of Family Medicine, University of Michigan Medical School, 1018 Fuller Street, Ann Arbor, MI 48104-1213 USA; University of Michigan Robert Wood Johnson Foundation Clinical Scholars Program, 2800 Plymouth Rd, Building 10, Room G016, Ann Arbor, MI 48109 USA; Department of Emergency Medicine, University of Michigan Medical School, 1500 East Medical Center Drive, Taubman Center B1-354, Ann Arbor, MI 48109 USA; Friends of Parkside, 5000 Conner Street, Detroit, MI 48213 USA; University of Michigan, 1018 Fuller Street, Ann Arbor, MI 48104 USA; Department of Internal Medicine, University of Michigan Medical School, 1500 East Medical Center Drive SPC 5352, Ann Arbor, MI 48109 USA; VA Health Services Research & Development Center of Excellence, Veterans Affairs Ann Arbor Healthcare System, 1500 East Medical Center Drive, Taubman Center B1-354, Ann Arbor, MI 48109 USA; Department of Research and Evaluation, Kaiser Permanente Southern California, 100 S. Los Robles, 2nd Floor, Pasadena, CA 91101 USA

**Keywords:** Text message, Survey, Community assessment, Low-income community, Community-based participatory research, Pilot study

## Abstract

**Background:**

It is not known whether using text messaging to administer real-time survey questions is feasible and acceptable among low-income, urban African American adults.

**Methods:**

We used a mixed methods approach including paper surveys, surveys administered by text message, and a focus group. Survey questions that included multiple choice, Likert-like scales, and open ended questions were administered by paper and sent via text message daily during varied times of day for six weeks.

**Results:**

In our study sample (n = 20), 90% of participants were female, and 100% were African American, with a median age of 30.7 years. Participants responded to 72% (1092/1512) of all multiple choice questions sent by text message and 76% (55/72) of the questions requiring responses on Likert-like scales. Content of responses on the paper and text message surveys did not differ. All participants reported in the focus group that they preferred text message surveys over other survey modalities they have used in the past (paper, phone, internet, in-person) due to ease and convenience.

**Conclusion:**

Text messaging is not only acceptable and feasible but is the preferred method of collecting real-time survey data in a low-income urban African-American community.

## Background

Text messaging is ubiquitous in the United States. As of May 2013, 91% of all U.S. adults owned a cell phone, and 79% of cell phone owners used it for text messaging [1]. Cell phone ownership among minorities and low-income Americans has rapidly increased over the past four years. For example, more black Americans own cell phones (93%) than white Americans (90%), and they are also more likely to use text messaging (79%) than white Americans (68%) [2].

In light of widespread cell phone use, corporations, organizations, and health care providers are exploring ways to use this technology to engage specific individuals [3–5]. Text messaging is being used to improve patient-provider communication in health care facilities, to deliver behavioral interventions, to prevent and help manage chronic conditions, and to promote healthy behaviors [6–8]. The use of text messaging in medical research has also shown promise. For example, text messaging has been used in studies to retain and engage study participants, test new interventions for chronic disease, and communicate with at-risk study participants [6, 9–11].

Despite widespread adoption of text messaging, to our knowledge no study has assessed the acceptability and feasibility of using text messaging to collect real-time data from low-income, inner city adults with the goal of informing community-based organizations on their current needs, opinions, and behaviors. The aim of this community-based participatory research (CBPR) study was to assess the acceptability and feasibility of using text messaging as a real-time survey tool in a low-income African American community in the eastside of Detroit.

## Methods

### Design

Our study used a mixed-methods community-based participatory research approach [12, 13] that included a paper survey, survey questions sent by text message, and a focus group. The two outcomes of interest in our study were: 1. Feasibility measured by successful administration of the text message survey questions and the overall response rate to these questions as well as during business hours (8 am to 3 pm) compared to non-business hours (4 pm to 11 pm and weekends); and 2. Acceptability measured by results of a focus group discussion designed to elicit participants’ opinions and perceptions around acceptability of text messaging as a community-based survey modality.

### Partnership

Our research team consisted of University of Michigan researchers, members of the Detroit Community-Academic Urban Research Center (URC), and representatives from Friends of Parkside (FOP). The URC is a community-based participatory research (CBPR) partnership that conducts research and implements interventions to promote health equity in the city of Detroit. FOP is a non-profit community organization in Detroit that provides supportive services to the residents of the Village at Parkside (TVP), a public housing complex on the eastside of Detroit with approximately 750 residents. The median income in TVP is between $16,000 and $26,000 with 32% to 47% living below the poverty line. African-Americans comprise over 90% of the population. A steering committee consisting of University of Michigan researchers, FOP community members and community partners was created. The steering committee met on a regular basis to design and plan the study, recruit and enroll participants, discuss data collection, and assess results. Data analysis was conducted by the University of Michigan researchers with frequent meetings with the full steering committee to discuss the interpretation and presentation of results.

Key principles of CBPR emphasizing equal partnership between all members and building capacity within communities guided this study from recruitment to data collection [12, 13]. Ethics approval for the study was obtained from the University of Michigan Institutional Review Board (Study eResearch ID HUM00065022, dated July 17, 2012).

### Recruitment and study sample

FOP maintains a database of approximately 200 client cell phone numbers for marketing community events. We used this database to send out a group text message with an invitation from the FOP office to join the study. We also distributed flyers throughout TVP and used word of mouth referrals as a recruitment modality. The FOP research assistant screened callers to ensure they met inclusion criteria. Individuals who were age 18 years or older, had a primary care doctor, and had text messaging capabilities were considered eligible and were invited to a recruitment meeting. Community members on the steering committee were excluded from the study.

Two recruitment meetings were held during which researchers explained the background and objectives of the study, collected demographic information, and obtained written informed consent. Data was then collected from enrolled participants in three ways: 1) a paper survey upon enrollment and at the midpoint of the study, 2) text message survey questions, and 3) a focus group also at the midpoint of the study.

### Study instruments

#### Paper survey

The first component of the paper survey consisted of 10 hypothetical scenarios of common primary care complaints based on the National Hospital Ambulatory Medical Care Survey of leading reasons for urgent outpatient medical visits [14]. We also included four anchoring questions that consisted of extreme scenarios, which were designed to prompt the participants to choose to go to the emergency department or stay home. For each question, participants were asked what kind of care they would seek, if any, based on the scenario and the time and date indicated. They then had the option of providing a free text explanation for why they made that choice. The three response choices for each question were “ER” if they would go to the emergency department, “MD” if they would seek advice from their primary care doctor’s office or “Nothing” if they would choose not to seek medical care.

The second component of the paper survey was administered at the midpoint of the study and consisted of questions assessing participants’ health literacy and health numeracy. These questions were added because of the poor grammar and spelling noted in the responses collected in the first half of the study. To assess health literacy, participants used a scale from 0 to 4 to answer Chew’s validated subjective test of health numeracy consisting of one question [15]. To assess health numeracy, participant’s used a scale from 1 to 6 to answer Zikmund-Fisher’s subjective three question test of health numeracy [16]. Please see “Survey questions texted to participants” section for a full list of questions.

**Survey questions texted to participants:**

**Hypothetical medical scenarios**

Your stomach has been hurting since last night. You threw up twice today.

You’ve had a sore throat for 4 days and feel sick.

You’ve felt sick and had a fever for 2 days.

You’ve had a cough, runny nose and headache for 3 days.

You’ve had a throbbing headache for 3 hours.

You have a red itchy rash on your legs, it has been there for 4 days.

You hurt your back picking up a child 2 days ago and it still hurts to move.

You slipped walking up the stairs and injured your knee. It is swollen and painful to walk.

You’ve had a runny nose for 5 days and now your right ear is hurting.

You slipped in the bathroom, injured your back, it hurts to lie down and when you bend over or twist.

You need a flu shot for your new job.

You have had a mole on your leg for 10 years and are now concerned it needs to be evaluated.

All of a sudden you can’t move your right arm or leg and you can’t speak normally.

You fell down the stairs your head is bleeding, you are confused and you can tell your leg is broken.

*Multiple choice response choices: ER = Emergency Department, MD = Primary care doctor, Nothing*

**Health Literacy and Health Numeracy Survey Questions**

How confident are you filling out forms Pick # 0 to 4. 0 Not at all. 1 A little bit. 2 Somewhat. 3 Quite a bit. 4 Extremely.

How good are you at working with fractions Pick # from 1 to 6. 1 Not at all good. 6 Extremely good.

How good are you at calculating a 15% tip Pick # from 1 to 6. 1 Not at all good. 6 Extremely good.

How good are you at working with percentages Pick # from 1 to 6. 1 Not at all good. 6 Extremely good.

#### Text message survey

Participants received two text message survey questions per day at different times of day for the next six weeks of the study period. These questions were identical to the questions asked on the paper survey. The text message surveys began four weeks after the initial paper survey to minimize recall of their original paper survey responses. Again, participants were asked to respond by text what kind of care they would seek, if any, based on the scenario and the time and date that they received the text message. Each question was pre-programed to be sent approximately six times regardless of their previous responses: twice during regular hours (8 am-4 pm), twice during off hours (5 pm-7 am) and twice during the weekend. The same brief health numeracy and literacy questions were also administered via text messaging once per day for four days at the end of the study period. All text message questions were sent and responses recorded through a secure online text messaging service (http://www.dialmycalls.com).

#### Focus group

A one-hour focus group was conducted by a research team member (WG) trained in focus group moderation to understand participants’ experiences, opinions, barriers and preferences in using text messaging as a survey tool. The focus group was performed at the midpoint of the study so that any technical difficulties that arose in the first half of the study could be addressed in the second half of the study. Due to significant participant challenges with transportation and scheduling (as is common with low-income participants), only one focus group was held. The moderator guide was constructed with the input of the community steering committee and also based on the participants’ response patterns and actual responses to the text message survey up to that point. For example, the focus group guide included questions regarding the timing and frequency of text messages because we found that participants were answering questions within the first several minutes of the question being sent or not at all. Please see Table 1 for a full summary of the content areas and sample questions included in the moderator guide.

**Table 1:** **Focus group content areas with sample questions**

CONTENT AREA	SAMPLE QUESTIONS
General questions	Tell me about your experiences these past 3 weeks answering text message survey questions. Do you think most people would be willing to answer questions through text messaging?
Timing and frequency	How do you feel about the number of texts you are getting each day? What number would be “just right”? What is the best time of day and day of the week to send you text message survey questions?
Technical issues	Tell me about your experiences receiving and sending text messages on your cell phone as part of the study. Does the type of phone you have or service you have affect your participation?
Text message surveys compared to other modes	How do you feel about answering survey questions on your cell phone versus other ways you have participated in surveys (on the phone, on paper, in person)?
Types of information and questions	What types of information would text message surveys be best at gathering? What kinds of questions would people be more willing to answer by text message?
Incentives	Tell me about the types (or amount) of incentives that would encourage or discourage you to respond to text questions?

#### Participant compensation

Participants received a maximum of $2 per text message ($1 for a multiple choice response and an additional $1 for a free text response) and $20 for each of the meetings they attended (one recruitment meeting, midpoint focus group, and the celebration meeting). All meetings were held at the FOP community center. A celebration meeting was held at the conclusion of the study to report preliminary results to the participants and community partners.

### Data analysis

#### Quantitative

Text message response rates were calculated for all the survey questions. The paper and text message surveys were then matched to compare the responses by the day of the week and time of day. Although this pilot study was designed to only examine feasibility and acceptability, a Chi-square test was used to identify any significant differences between the text message and paper survey responses. STATA 12.1 software was used for the data analysis. (Stata Corp.; College Station, TX).

#### Qualitative

The focus group was transcribed verbatim by a professional transcriptionist. Four research team members on the steering committee who were present during the focus group reviewed the transcript for accuracy (TC, AS, EC, WG). We used a general inductive approach informed by thematic analysis [17–19]. The transcript was reviewed line by line by the same four researchers to identify prominent concepts and ideas to draft preliminary coding categories. These initial findings were reviewed, coding categories were created, and clarified as a team. We then engaged in an inductive process of reading and manually coding the transcript together. Codes were further clarified and a codebook with definitions was developed. From this codebook, the entire transcript was coded independently. Inter-coder agreement was 92%. Data was reviewed in frequent meetings and discussions, using memos to identify emerging themes and describe relationships among coding categories [20]. The final coding scheme and analysis of the findings were reviewed, and disagreements were discussed until consensus was reached between all four researchers. We organized the results using the coding scheme structure and illustrated the themes with representative quotations. To increase the validity of our qualitative data, main ideas were summarized and clarified during the focus group and “member checking” was performed where the overall results of the study were presented to each participant at the post-study celebration. Each participant indicated that the results included and accurately represented their viewpoints. Four researchers performed the analysis of the qualitative data, though preliminary results were shared with the full steering committee including community members throughout the process during biweekly research meetings.

## Results

### Demographics

The sample in this pilot study consisted of 20 community members. The demographic characteristics of the participants are described in Table 2. Eighteen participants (90%) completed the study. Two participants lost service on their cell phones due to non-payment and were not able to receive text messages during the study period.

**Table 2:** **Characteristics of study population, n = 20**

**Age (Years)**	
Range	19 - 62
Median	30.7
**Females**	17 (85%)
**Race/Ethnicity**	
Black	20 (100%)
**Education**	
<HS	4 (20%)
HS Grad or Equivalent (GED)	3 (15%)
Some College or College Grad	13 (65%)
**Work status**	
Student	4 (20%)
Employed	7 (35%)
Unemployed	9 (45%)
**Type of phone**	
Touch Screen/Keyboard	19 (95%)
Number keypad only	1 (5%)
**Texting plan**	
Unlimited	18 (90%)
Other	2 (10%)

HS = High School.

GED = General Educational Development.

### Text message survey

Participants responded to 72% (1092/1512) of all multiple choice questions sent by text message and 76% (55/72) of the literacy/numeracy questions requiring responses on Likert-like scales (Table 3). The average response rate by participant was 72% (median 83%, range 2-99%). Among the medical scenario questions, 95% of participants provided free text responses along with multiple choice responses. Participants used common abbreviations and slang in their free text responses (Example: “Er why u slip in tub an u cud of broke anythin so yup er” “Nothin cuz a asprin will do da trick”). Spelling was often incorrect, though usually easy to decipher (“Er because it mite be broken” “Nothing cuz over da corner medicine will do”). Seventy-five percent of participants texted back within the first 29 minutes if they responded at all. The median response time was five minutes (range 0-905 minutes). Response rate during business hours was 72.0% with a median response time of six minutes (mean 30.9 minutes, range 0-747 minutes) compared to 72.4% with a median response time of four minutes (mean 29.3 minutes, range 0- 905 minutes) during evening hours and weekends. There was no statistically significant difference between response rates during business hours compared to evening and weekend hours. There was also no significant difference between responses given by text message and those given on paper, though our study was not powered or designed to detect differences.

**Table 3:** **Response rate and response time of text survey questions n = 18**

	Questions sent	Responses	Response rate	With free text	Median time to response (minutes)	Range (minutes)
**Medical scenarios**	1512	1092	72.2%	94.9%	5	0-905
Business Hours	671	483	72.0%	92.1%	6	0-747
Evening/Weekend Hours	841	609	72.4%	96.7%	4	0-905
**Health literacy/numeracy questions**	72	55	76.4%			

Business Hours: Text questions sent between 8 am-3 pm.

Evening/Weekend Hours: Text questions sent between 4 pm-11 pm.

With free text =% of answered multiple choice text message survey question where participants also included a free text response.

### Focus group

Twelve participants participated in the focus group and several major themes emerged from the focus group discussions (Table 4). Overall, participants expressed largely positive views regarding text message surveys, stating that they are “plain and simple”. They reported more willingness to answer a survey through text messaging, stating, “[I] would read a text [survey] faster than if somebody sent me a survey through the mail”. Texting was seen as a common and frequent form of communication, with one participant stating, “That’s all people do is text anyway!” Other participants stated that texting allows them to communicate in their “own language”. All participants explicitly reported that they preferred text message surveys over paper, phone, in-person, and Internet surveys they have done in the past due to ease and convenience. These positive sentiments were universal among all participants regardless of age.

**Table 4:** **Focus group themes - exploring text messaging as a survey tool in a low income community**

Category	Theme	Representative quote
**General experience with text survey**	Positive	“I would read a text [survey] faster than if somebody sent me a survey through the mail”.
		“That”s all people do is text anyway!”
		“It is plain and simple. It isn’t like you are sending off paragraphs at a time.”
	Negative	“But I would forget. You know, once you get to your destination you forget [to answer the text]”.
**Technical issues**	Phone service provider issues	“Like sometimes in my house, in our neighborhood the reception is bad so if a text comes through and I go outside I will respond to the text and like the next day I go and see that it was saved in ‘address’ like, you didn’t send”.
**Timing and frequency**	Number of texts	“Two [a day] is enough for me”.
		“Send some more! Send five a day!”
	Timing	“I didn’t like the ones that came after ten because I turn in like at eight. So the ones I got at ten o’clock you might have got a weird answer”.
		“Sundays are not good because of church”.
**Texts compared to other modes**	Preferred over paper, phone, face-to-face, internet surveys	“I want to do more text surveys”.
		“I like text surveys better than those other kinds [of surveys]”.
		“It takes a shorter time if I text than just writing it on a sheet of paper”.
		“It’s a lot quicker than taking a survey on the internet. I will tell you that!”
**Implementation for text surveys**	Types of survey questions	“Text would not be good for sensitive stuff”.
		“If I thought it was going to be that personal I would say one on one [interviewing] is better”.
	Incentives	“I think you would have to put it out there in the beginning that it is a quarter, but I would do it [answer the text survey]”.
		“I would do it for nothing if it’s going to help people in the long run with their insurance because I don’t have none [insurance]”.

One participant expressed concerns over cell phone reception as a barrier to responding to text message surveys, though all other participants reported that they had no logistical problems in responding to the text survey questions.

In regards to timing and frequency, most participants felt that two questions per day was sufficient. Some participants, however, felt that they could answer up to five text message survey questions per day. Participants disliked when texts were sent early in the morning, late in the evening, during church (Sunday morning) or while they were in class. One participant stated, “I didn’t like the ones that came after 10 because I turn in like at eight. So the ones I got at ten o’clock you might have got a weird answer”.

Some participants stated that they would prefer in-person communication for sensitive information, stating “text would not be good for sensitive stuff”. Others felt comfortable texting even about sensitive topics.

In regards to incentives, participants did not have a strong sense of how much would be appropriate. However, several participants said they would answer text message survey questions for as little as 25 cents per text response. Another participant stated, “I would do it for nothing if it’s going to help people in the long run,” representing the common sentiment that they would participate for free as long as the study was for a good cause.

Results of the hypothetical medical scenario questions are presented in another manuscript [21]. This article focuses solely on the feasibility and acceptability of text messaging as a survey modality.

## Discussion

Text messaging is a feasible and acceptable survey tool to gather real-time data from low-income, inner-city community members. Our findings are consistent with studies among other populations in other settings that have found that text messaging is a reliable, valid and feasible research tool [22, 23]. However, our study adds to this body of literature by finding that text messaging can also be easily and inexpensively used by community-based organizations to gather information in a very short time regarding the preferences, opinions, and needs of their community. Using a publically available website, administration of text survey questions cost less than $50 for a month of unlimited texting and data collection. Furthermore, participants in our study reported in the focus group that they preferred this modality over traditional forms of survey data collection they have used in the past (paper, phone, face-to-face, internet).

Our study has several important implications for community-based survey research and as a tool for community-based organizations (CBOs). We found that all three types of text message survey questions studied were feasible and acceptable in our sample of community members: questions with multiple choice responses; scaled responses; and open-ended responses. The series of questions consisting of hypothetical medical scenarios all had the same multiple choice responses (ER, MD, Nothing) as well as the opportunity to free text. Participants were quite willing to give free text responses as demonstrated by the high percentage of participants who consistently provided free text responses (95%). Of note, participants did receive an additional one-dollar incentive for free text responses. Based on their free text responses, we were able to gain a great deal of contextual data on respondents’ thought processes and beliefs described in their own words. Despite the use of abbreviations and misspellings, their free text responses were easy to decipher and analyze using qualitative methods.

The health literacy and numeracy survey questions asked participants to answer on a Likert-like scale of 0 to 4 or 1 to 6. Although the scale was presented in a text message only and was not explained in person, participants reported that these responses were easy to understand and select a choice. Our pilot study demonstrates that text messaging these questions is feasible and acceptable; however, larger studies are warranted to determine whether health literacy and numeracy testing by text messaging is valid. If so, CBOs and researchers could use this method to quickly and inexpensively tailor their interventions, communications, and policies for communities of varying health literacy and numeracy levels.

Another important implication of our study is the potential utility of text message surveys sent by CBOs to quickly gather real-time information from community members concerning community needs and issues. In the focus group, it was clear that older participants were equally facile with texting as younger participants. Participants reported that text message survey questions were easy to read and understand due to their brevity and simple wording, potentially reducing the barrier of low literacy. Participants were also able to answer text questions during a time and place that was convenient for them, which could improve response rates. Interestingly, participants said that texting allowed them to respond in their own “language”. Many also stated that they use it daily as their primary form of communication with friends and family members. Perhaps by using this familiar modality, participants are able to give more nuanced and accurate answers.

### Limitations

Limitations of this pilot study are primarily related to the small sample size. Participants were recruited from one urban, low-income community in Detroit, MI, which may limit generalizability. However, our findings are likely to be similar to other urban low-income communities with similar demographics. Future studies should not only have a larger sample size, but also include greater diversity in ethnicity, socio-economic status, and geography to have greater generalizability. Also, the survey questions in our study were specifically focused on participants’ usage of healthcare services and a cash incentive was provided for responses. Participation and response rates may vary with differing topics and the amount and form of incentive offered.

## Conclusions

Assessing the real-time needs and preferences of communities can be logistically difficult, time consuming, and often expensive. By using text messaging as a survey tool, CBOs and health care workers have the potential of gathering real-time information accurately, quickly, and inexpensively. Our study begins to show the potential of text messaging in giving more community members a voice as well as the potential to empower and engage more individuals in the activities and issues involving their community. Text messaging thus taps into the rich human capital in communities in a way that is convenient for both community members as well as CBOs. Our findings show that text messaging is not only acceptable and feasible, but is the preferred method of collecting real-time survey data in a low income community.
